# Outpatient Surgery and Unplanned Overnight Admission in Bilateral Inguinal Hernia Repair: A Population-based Study

**DOI:** 10.1007/s00423-024-03358-0

**Published:** 2024-05-27

**Authors:** Nils Jimmy Hidalgo, Salvador Guillaumes, M. Magdalena Llompart-Coll, Paula González-Atienza, Irene Bachero, Dulce Momblán, Óscar Vidal

**Affiliations:** 1 Department of Gastrointestinal Surgery, Institute of Digestive and Metabolic Diseases, Hospital Clínic Barcelona, C. de Villarroel, 170, Barcelona, 08036 Spain; 2 Department of General and Digestive Surgery, Institute of Digestive and Metabolic Diseases, Hospital Clínic Barcelona, Barcelona, Spain

**Keywords:** Bilateral inguinal hernia, Outpatient surgery, Unplanned overnight admission, Laparoscopic surgery

## Abstract

**Purpose:**

The use of outpatient surgery in inguinal hernia is heterogeneous despite clinical recommendations. This study aimed to analyze the utilization trend of outpatient surgery for bilateral inguinal hernia repair (BHIR) in Spain and identify the factors associated with outpatient surgery choice and unplanned overnight admission.

**Methods:**

A retrospective observational study of patients undergoing BIHR from 2016 to 2021 was conducted. The clinical-administrative database of the Spanish Ministry of Health RAE-CMBD was used. Patient characteristics undergoing outpatient and inpatient surgery were compared. A multivariable logistic regression analysis was performed to identify factors associated with outpatient surgery choice and unplanned overnight admission.

**Results:**

A total of 30,940 RHIBs were performed; 63% were inpatient surgery, and 37% were outpatient surgery. The rate of outpatient surgery increased from 30% in 2016 to 41% in 2021 (*p* < 0.001). Higher rates of outpatient surgery were observed across hospitals with a higher number of cases per year (*p* < 0.001). Factors associated with outpatient surgery choice were: age under 65 years (OR: 2.01, 95% CI: 1.92–2.11), hospital volume (OR: 1.59, 95% CI: 1.47–1.72), primary hernia (OR: 1.89, 95% CI: 1.71–2.08), and laparoscopic surgery (OR: 1.47, 95% CI: 1.39–1.56). Comorbidities were negatively associated with outpatient surgery. Open surgery was associated (OR: 1.26, 95% CI: 1.09–1.47) with unplanned overnight admission.

**Conclusions:**

Outpatient surgery for BHIR has increased in recent years but is still low. Older age and comorbidities were associated with lower rates of outpatient surgery. However, the laparoscopic repair was associated with increased outpatient surgery and lower unplanned overnight admission.

**Supplementary Information:**

The online version contains supplementary material available at 10.1007/s00423-024-03358-0.

## Introduction

Inguinal hernia is a common surgical issue accounting for 75% of all abdominal wall hernias [1] and Inguinal hernia repair ranks among the most frequently conducted surgical interventions worldwide [2, 3]. As such, changes in the type of hospitalization, utilization of minimally invasive surgical techniques, and rate of postoperative complications of inguinal hernia surgery can significantly impact the healthcare system.

Advances in surgical and anesthetic techniques have increased the proportion of inguinal hernia repairs conducted as outpatient surgery [4]. Outpatient surgery offers several advantages, including early mobilization, increased patient satisfaction, decreased susceptibility to nosocomial infections and venous thromboembolism, and minimized costs associated with conventional inpatient care [5–8].

Nevertheless, the outpatient inguinal hernia repair rate remains variable despite its wide acceptance and international clinical guideline recommendation [3, 9]. Patient characteristics, surgeon preferences, and health system incentives influence its choice. It is recommended that over 70% of adult inguinal hernia repairs should be conducted as outpatient surgery [10–12].

Some authors suggest that bilateral inguinal hernias should preferably receive hospital treatment due to the increased risk of perioperative complications [13, 14]. In addition, bilateral inguinal hernia has been described as a predictor of ambulatory failure [14]. However, according to other studies, bilateral inguinal hernia is not a limitation for outpatient surgery and is not associated with an increased risk of failure [15–17]. Limited research exists on outpatient surgery utilization for inguinal hernia, and no specific studies exist on bilateral inguinal hernia.

This study aimed to analyze the utilization trend of outpatient surgery for BHIR in Spain, identify the factors associated with the choice of outpatient surgery, and the factors associated with unplanned overnight admission in patients initially scheduled for outpatient surgery.

## Materials and Methods

### Study Design

A retrospective observational study was conducted using the Hospital Discharge Registry of the Spanish Ministry of Health (Registro de Actividad de Atención Especializada-Conjunto Mínimo Básico de Datos, RAE-CMBD) [18]. In Spain, the RAE-CMBD is a mandatory registry of the diagnoses and healthcare procedures performed in all public and private hospitals nationwide, using the International Classification of Diseases Version 10 (ICD-10) codes. The data is obtained from the information in each patient’s discharge report. It records three types of variables: patient identification, identification of the care episode, and clinical variables. The physician completes the hospital discharge information in the discharge report, and subsequently, the health coding specialist performs the coding of the information contained in the hospital discharge report.

### Study Population

**Fig. 1:**
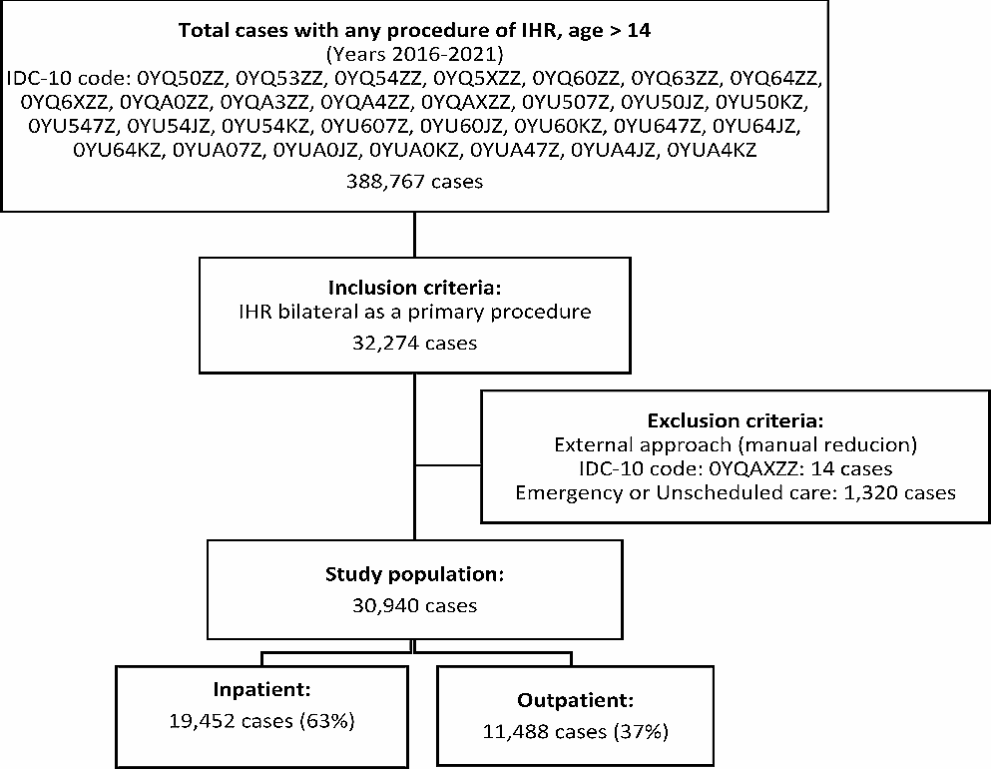
Case selection flow chart. IHR: Inguinal hernia repair. IDC-10: 10th revision of the international statistical classification of diseases

Our study included patients who underwent BIHR in the Spanish National Health System hospitals from 2016 to 2021. The flowchart (Fig. 1) shows the ICD-10 diagnostic codes used to identify patients.

Inclusion criteria: (1) Patients with main BIHR procedure and (2) Age greater than or equal to 15 years.

Exclusion criteria: (1) Manual reduction of the hernia and (2) Emergency or unscheduled care.

### Variables Analyzed

#### Demographic Characteristics and Comorbidities

Data analysis included age, sex, and comorbidities. The specific comorbidities were identified using ICD-10 diagnostic codes described by Quan et al. [19]. The specific comorbidities considered included arterial hypertension, heart disease, chronic lung disease, renal disease, liver disease, diabetes mellitus, obesity, peripheral vascular disease, cerebrovascular disease, rheumatic disease, and alcohol and tobacco abuse. Charlson and Elixhauser comorbidity indices were calculated for each patient. Supplementary material presents the ICD-10 codes used for comorbidities.

#### Hospital Volume

The study analyzed the relation between the outpatient surgery rate and hospital volume, which was defined as the number of BIHRs performed by year.

#### Characteristics of the Hernia and Surgery

The pre-surgical presence of recurrent hernia and complicated hernia was recorded. The complicated hernia was defined as obstruction or gangrene in the diagnostic code. The surgical approach, open or laparoscopic, was recorded.

#### Outpatient Surgery

Patients scheduled for inpatient and outpatient surgery for BIHR were identified. We used the variable “type of contact” from the RAE-CMBD database to identify patients initially scheduled for outpatient surgery. A comparative analysis between the two groups was conducted, and a multivariable analysis was performed to determine the factors associated with the choice of outpatient surgery.

#### Unplanned Overnight Admission

The unplanned overnight admission and unplanned readmission rate to the hospital are quality markers for ambulatory surgery units. Patients who required unplanned overnight admission among those initially scheduled for outpatient surgery were identified. Unplanned overnight admission was defined as a hospital stay lasting ≥ 1 day.

### Statistical Analysis

The Chi-square test was used for the qualitative variables. For quantitative variables with normal distribution, Student’s t-test was used to compare between two groups. For non-normal distributions, the non-parametric Mann-Whitney U test was used.

The Cochran-Armitage test was used for trend analysis of ordinal categorical variables,

A multivariable logistic regression analysis was performed to identify factors associated with the choice of outpatient surgery and to identify factors associated with unplanned overnight admission.

Statistical significance was set at *p* < 0.05. IBM SPSS 27.0 software (Armonk, NY: IBM Corp) was used for statistical analysis.

### Ethical Aspects

The analyzed data is anonymous and sourced from a database under the management of the Spanish Ministry of Health, adhering to the data protection regulations in Spain. Identifying patients at the individual or reporting unit level is impossible, and using information from clinical-administrative bases does not require the approval of a Medical Research Ethics Committee.

## Results

### Utilization Trend of Outpatient Surgery in BIHR

**Fig. 2:**
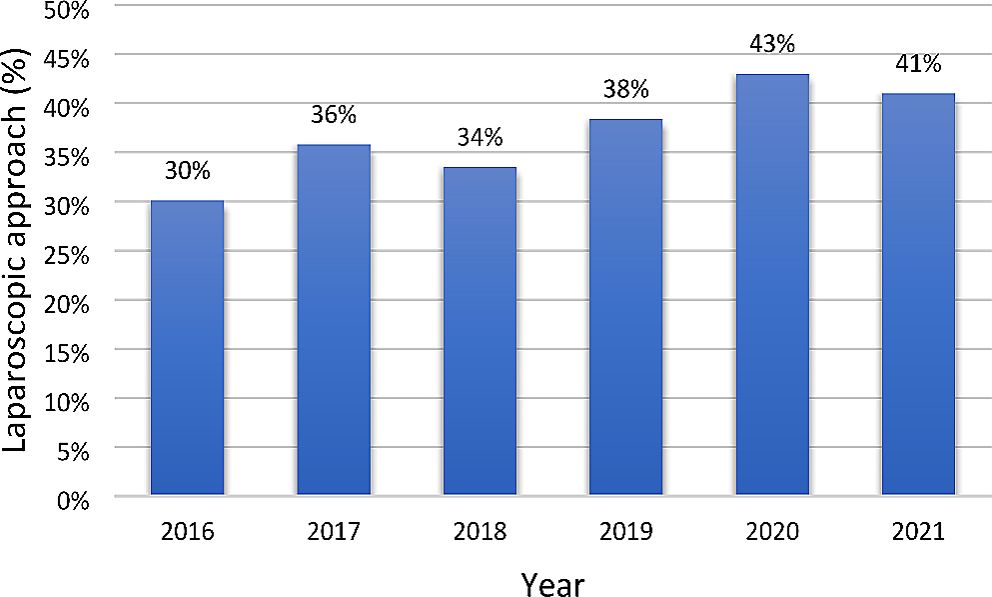
The outpatient surgery rate in bilateral inguinal hernia repair. Cochran-Armitage test for trend was significant (*p* < 0.001)

Our study included 30,940 BIHR, 19,452 (63%) as inpatient surgeries, and 11,488 (37%) as outpatient surgeries. Over the analyzed period, there was a statistically significant upward trend in the utilization of outpatient surgery in the test of Cochran-Armitage (*p* < 0.001), increasing from 30% in 2016 to 41% in 2021 (Fig. 2).

### Hospital Volume

**Fig. 3:**
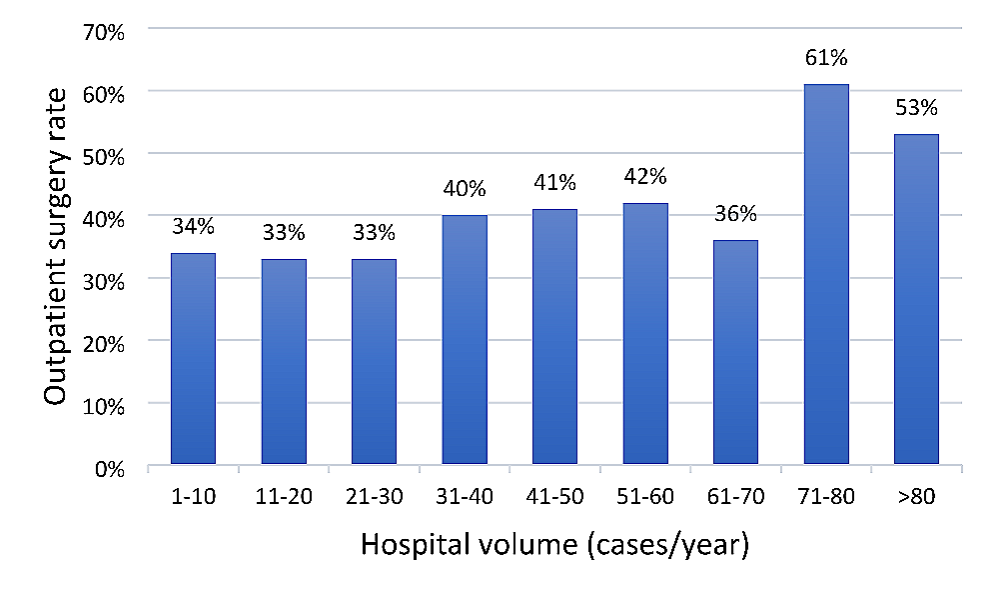
The outpatient surgery rate of bilateral inguinal hernia repair by hospital volume. Cochran-Armitage test for trend was significant (*p* < 0.001)

The outpatient surgery utilization rate was proportionally higher (*p* < 0.001) in hospitals with the highest number of BIHRs performed per year (Fig. 3).

### Demographic Characteristics and Comorbidity

The mean age was higher in the inpatient surgery group than in the outpatient surgery group (63.58 ± 13.29 vs. 58.22 ± 12.99, *p* < 0.001), and there were no significant differences in sex between the two groups (Table 1). The comorbidities were higher in inpatient surgery group with a significant difference (*p* < 0.001).

**Table 1:** Characteristics of the patients with bilateral inguinal hernia repair (2016–2021)

	Total *N* = 30,940	Inpatient *N* = 19,452	Outpatient *N* = 11,488	*p*-value
Age, Mean ± SD	61.59 ± 13.43	63.58 ± 13.29	58.22 ± 12.99	< 0.001
Age < 65 years, N (%)	17,285 (55.9)	9,652 (49.6)	7,633 (66.4)	< 0.001
Age ≥ 65 years, N (%)	13,655 (44.1)	9,800 (50,4)	3,855 (33.6)	< 0.001
Sex, N (%)				0.942
Male	28,682 (92.7)	18,034 (92.7)	10,648 (92.7)	
Female	2,258 (7.3)	1,418 (7.3)	840 (7.3)	
Comorbidities, N (%)				
Arterial hypertension	8,030 (26)	6,594 (33.9)	1,436 (12.5)	< 0.001
Heart disease	2,448 (7.9)	2,203 (11.3)	245 (2.1)	< 0.001
Chronic pulmonary disease	1,874 (6.1)	1,543 (7.9)	331 (2.9)	< 0.001
Renal disease	559 (1.8)	496 (2.5)	63 (0.5)	< 0.001
Liver disease	517 (1.7)	410 (2.1)	107 (0.9)	< 0.001
Diabetes mellitus	2,606 (8.4)	2,137 (11)	469 (4.1)	< 0.001
Obesity	857 (2.8)	649 (3.3)	208 (1.8)	< 0.001
Peripheral vascular disease	387 (1.3)	355 (1.8)	32 (0.3)	< 0.001
Cerebrovascular disease	162 (0.5)	142 (0.7)	20 (0.2)	< 0.001
Rheumatic disease	240 (0.8)	198 (1)	42 (0.4)	< 0.001
Alcohol abuse	640 (2.1)	491 (2.5)	149 (1.3)	< 0.001
Tobacco use	3,883 (12.6)	2893 (14.9)	990 (8.6)	< 0.001
Charlson Index, Mean (SD)	0.3 ± 0.76	0.4 ± 0.89	0.12 ± 0.47	< 0.001
Elixhauser Index, Mean (SD)	0.76 ± 2.75	1.07 ± 3.17	0.23 ± 1.71	< 0.001
Hernia characteristics, N (%)				
Recurrent hernia	2,271 (7.3)	1,712 (8.8)	559 (4.9)	< 0.001
Primary hernia	28,669 (92.7)	17,740 (91.2)	10,929 (95.1)	< 0.001
Surgery approach, N (%)				< 0.001
Open surgery	24,524 (79.3)	15,887 (81.7)	8,637 (75.2)	
Laparoscopy surgery	6,416 (20.7)	3,565 (18.3)	2,851 (24.8)	
Hospital volume, N (%)				< 0.001
1–20 cases/year	10,795 (34.9)	7,205 (37)	3,590 (31.3)	
20–40 cases/year	12,907 (41.7)	8,170 (42)	4,737 (41.2)	
40–60 cases/year	4,462 (14.4)	2,617 (13.5)	1,845 (16.1)	
60–80 cases/year	863 (2.8)	560 (2.9)	303 (2.6)	
>80 cases/year	1,913 (6.2)	900 (4.6)	1,013 (8.8)	

*SD: standard deviation*

### Characteristics of the Hernia and Surgery

The proportion of recurrent hernia was higher in the inpatient surgery group (*p* < 0.001). The use of laparoscopic surgery was higher in the outpatient surgery group than in the inpatient surgery group (24.8% vs. 18.3%, *p* < 0.001).

### Factors Associated with the Use of Outpatient Surgery

In the multivariable logistic regression analysis, the factors independently associated with the choice of outpatient surgery were: age under 65 years (OR: 2.01, 95% CI: 1.92–2.11), hospital volume > 60 cases/year (OR: 1.59, 95% CI: 1.47–1.72), primary inguinal hernia (OR: 1.89, 95% CI: 1.71–2.08), and laparoscopic surgery (OR: 1.47, 95% CI: 1.39–1.56). The comorbidities presented a negative association with the choice of outpatient surgery (Table 2).

**Table 2:** Univariable and multivariable analysis of factors associated with the choice of outpatient bilateral inguinal hernia repair

	Univariable analysis		Multivariable analysis	
	OR (95% CI)	p-value	OR (95% CI)	p-value
Age < 65 years	2.01 (1.92–2.11)	< 0.001	1.34 (1.27–1.41)	< 0.001
Sex Male	1.01 (0.92–1.09)	0.942		
Hospital volume > 60 cases/year	1.59 (1.47–1.72)	< 0.001	1.59 (1.46–1.72)	< 0.001
Primary hernia	1.89 (1.71–2.08)	< 0.001	1.78 (1,61-1.97)	< 0.001
Arterial hypertension	0.28 (0.27–0.29)	< 0.001	0.41 (0.38–0.44)	< 0.001
Heart disease	0.17 (0.15–0.19)	< 0.001	0.31 (0.29–0.36)	< 0.001
Chronic pulmonary disease	0.34 (0.31–0.39)	< 0.001	0.51 (0.45–0.58)	< 0.001
Renal disease	0.21 (0.16–0.27)	< 0.001	0.59 (0.45–0.78)	0.001
Liver disease	0.44 (0.35–0.54)	< 0.001	0.59 (0.47–0.73)	< 0.001
Diabetes mellitus	0.35 (0.31–0.38)	< 0.001	0.64 (0.58–0.72)	< 0.001
Obesity	0.53 (0.46–0.63)	< 0.001	0.79 (0.67–0.93)	0.005
Peripheral vascular disease	0.15 (0.11–0.22)	< 0.001	0.35 (0.24–0.51)	< 0.001
Cerebrovascular disease	0.24 (0.15–0.38)	< 0.001	0.37 (0.32–0.86)	0.01
Rheumatic disease	0.53 (0.26–0.49)	< 0.001	0.53 (0.37–0.75)	< 0.001
Laparoscopic surgery	1.47 (1.39–1.56)	< 0.001	1.31 (1.24–1.39)	< 0.001

*OR: odds ratio, CI: confidence interval*

### Unplanned Overnight Admission

Among the 11,488 patients initially scheduled for outpatient surgery, 1143 (9.9%) required an unplanned overnight admission. In this study, open surgery was independently associated (OR: 1.26, 95% CI: 1.09–1.47) with unplanned overnight admission, while hospital volume > 60 cases/year was negatively associated (OR: 0.16, 95% CI: 0.11–0.24) with unplanned overnight admission (Table 3).

**Table 3:** Univariable and multivariable analysis of factors associated with unplanned overnight admissions of outpatient bilateral inguinal hernia repair

	Univariable analysis		Multivariable analysis	
	OR (95% CI)	p-value	OR (95% CI)	p-value
Age ≥ 65 years	0.91 (0.79–1.03)	0.137		
Sex Male	0.92 (0.72–1.17)	0.504		
Hospital volume > 60 cases/year	0.16 (0.11–0.24)	< 0.001	0.17 (0.11–0.24)	< 0.001
Recurrent hernia	1.2 (0.92–1.57)	0.175		
Arterial hypertension	1.49 (0.94–2.38)	0.091		
Heart disease	1.27 (0.86–1.87)	0.226		
Chronic pulmonary disease	0.89 (0.62–1.32)	0.585		
Renal disease	0.15 (0.02–1.05)	0.056		
Liver disease	0.93 (0.49–1.79)	0.834		
Diabetes mellitus	0.79 (0.56–1.11)	0.173		
Obesity	0.75 (0.45–1.26)	0.274		
Peripheral vascular disease	0.29 (0.04–2.14)	0.225		
Cerebrovascular disease	0.48 (0.07–3.56)	0.469		
Rheumatic disease	0.95 (0.34–2.67)	0.926		
Open surgery	1.26 (1.09–1.47)	0.002	1.23 (1.06–1.43)	0.007

*OR: odds ratio, CI: confidence interval*

## Discussion

The use of outpatient surgery for BIHR has increased in recent years. Factors such as age below 65 years, larger hospital volume, primary inguinal hernia, and laparoscopic approach were associated with the choice of outpatient surgery. Conversely, comorbidities showed a negative association. Furthermore, open surgery was independently linked to unplanned overnight admission.

The advantages of outpatient inguinal hernia surgery are widely recognized, including higher patient satisfaction rates and reduced costs [7, 20]. Hospital-admitted inguinal hernia surgery costs 56% more than outpatient surgery [6, 21]. Additionally, it enables better utilization of hospital resources by ensuring beds are available for patients with more severe conditions.

The utilization of outpatient surgery for inguinal hernia repair has increased globally [22, 23]. However, the proportion of use varies significantly across countries. Some countries have achieved high utilization rates exceeding 70%. Rates of 70% were reported in Denmark [24] and 74% in France [14]. In a study conducted in hospitals in northeast Italy, the rate was even higher at 76% [4]. While in other countries, such as Germany, the proportion of outpatient surgery in inguinal hernia repair was only 14% in 2019 [13]. A recent study in Spain reported that 54% of all inguinal hernia repairs were performed as outpatient procedures [25]. The variability in outpatient surgery utilization can be attributed to differences in patient selection criteria and economic incentives for hospitals and surgeons to promote outpatient surgeries [13]. The number of procedures performed by outpatient surgery in Spain could be greater through the application of measures by the National Health System that encourage hospitals to increase the use of outpatient surgery to optimize public health resources. In our analysis of bilateral hernias, we observed an increasing trend in outpatient surgery, from 30% in 2016 to 41% in 2021. In 2020, we observed an increase in the use of outpatient surgery up to 43%, probably influenced by the COVID-19 pandemic. However, the use of outpatient surgery in 2021 was 41%. Future studies are necessary to analyze whether the trend toward increased use of outpatient surgery observed during the year of the COVID-19 pandemic will continue in the coming years.

The patient’s age has been considered by some authors as a criterion for selecting candidates for outpatient surgery in inguinal hernia repair [26, 27]. Our study found that age under 65 was independently associated with the choice of outpatient surgery. However, other studies have shown that older patients do not have higher complication rates than younger patients [8, 28, 29], suggesting that age should not be a contraindication for outpatient surgery [30, 31]. Including elderly patients in outpatient surgery can increase utilization rates and provide them with the benefits of a shorter hospital stay, such as reduced cognitive impairment [32].

Our study found that comorbidities were negatively associated with the choice of outpatient surgery for BIHR. The Spanish Ministry of Health uses the classification of the American Society of Anesthesiologists (ASA) [33] in its recommendations for selecting candidate patients for outpatient surgery. It considers suitable patients with ASA 1, ASA 2, and ASA 3 without decompensation. However, studies conducted in inguinal hernia surgery suggest that comorbidities or the ASA score should not be a contraindication for outpatient surgery [34, 35]. Utilizing outpatient surgery in patients with comorbidities could offer them the advantages of reduced risk for nosocomial infections and venous thromboembolic complications, to which they are more vulnerable [5, 7, 8].

The characteristics of the hernia can play a role in determining the suitability of outpatient surgery. Strangulated hernia and large inguinoscrotal hernia have been considered exclusion criteria in some studies [36]. However, some authors argue that recurrent hernia should not be a contraindication for outpatient surgery [14, 17]. In our study, we found that recurrent hernia was negatively associated with the choice of outpatient surgery.

Hospitals with higher case volumes and experienced surgeons tend to have shorter surgical times and lower complication rates [37]. These favorable outcomes are conducive to the implementation of outpatient surgery. In our study, we found that hospitals with a higher number of cases performed annually were more likely to choose outpatient surgery.

Decreased postoperative pain is one of the main factors that favor outpatient surgery [38], and it is known that laparoscopic inguinal hernia repair is associated with reduced postoperative pain, faster recovery, and fewer complications [39–42]. However, the utilization rates are variable: 61% in Denmark [43], 38% in the USA [44], 23% in England [45] and 5.7% in Spain [46]. The high rate of use of laparoscopy in countries such as Denmark, where it reaches up to 96% in bilateral hernias [47], could be related to the high rates of use of outpatient surgery. The benefits of laparoscopic are greater in a bilateral inguinal hernia, and international clinical guidelines recommend performing BIHR using a laparoscopic approach [3, 48–51]. Our study observed that laparoscopic repair was independently associated with the choice of outpatient surgery. However, despite these advantages, the utilization rate of laparoscopic bilateral inguinal hernia repair in Spain remains low, with a reported rate of 23% in 2019 [52]. Efforts to increase the laparoscopic BIHR rate could increase outpatient surgery utilization in these patients. Furthermore, although the higher cost of laparoscopic surgery is a limiting factor [53], the cost-effectiveness of outpatient surgery would offset this expense.

The rate of unplanned admissions in ambulatory inguinal hernia surgery varies considerably in the literature, ranging from 0 to 19% [14, 15, 30, 54–57]. In our study, we observed an unplanned overnight admission rate of 9.9%. Several published studies have been performed to identify predictors of unplanned admission to improve the outcomes of outpatient surgery for inguinal hernia. Some have identified ASA grades 3 and 4 as predictors of unplanned admission [14, 54, 56]. Other studies found that older patients, body mass index greater than 30, spinal anesthesia, and longer duration of surgery are factors that predict unplanned admission [14, 35, 54]. However, in our study, comorbidities and age greater than or equal to 65 years were not associated with unplanned overnight admission. Similar findings have been reported, where other authors have also found no association between comorbidities [35] and older age [14] with outpatient procedure failure. Our study observed that higher hospital volume (number of cases per year) was associated with decreased unplanned overnight admissions. This can be explained because more experienced surgeons achieve shorter procedure times and a lower rate of complications, which reduces the probability of unplanned overnight admissions.

In the multivariable analysis, open surgery was independently associated with unplanned overnight admission. Therefore, a transition to laparoscopic surgery of the BIHR could increase the use of outpatient surgery and decrease the rate of unplanned admissions. This transition can be achieved safely and feasibly through a structured and systematic training process without an increase in complication or recurrence rates [58].

Our study has limitations inherent to clinical-administrative databases, including the absence of clinical data such as body mass index, surgical technique, surgical duration, reasons for unplanned overnight admission, and post-discharge clinical outcomes such as unplanned readmission to the hospital. Furthermore, potential underreporting may exist due to incomplete discharge reports or errors made during data recording by technical-administrative staff. However, the main strength of our study is its statistical power due to the large sample size. Previous studies have demonstrated the usefulness of clinical-administrative databases such as the RAE-CMBD for surgical research [46, 59–62].

## Conclusions

The use of outpatient surgery for bilateral inguinal hernia in Spain has recently increased, although it remains low. Older age and comorbidities were associated with less use of outpatient surgery. However, laparoscopic repair was associated with increased outpatient surgery and a reduced rate of unplanned overnight admissions. Adopting less restrictive inclusion criteria and a transition to laparoscopic bilateral inguinal hernia repair could increase the rate of outpatient surgery.

### Electronic Supplementary Material

Below is the link to the electronic supplementary material.

Supplementary Material 1

## Data Availability

The datasets used and analyzed during the current study are available from the corresponding author on reasonable request.

## References

[1] Kingsnorth A, LeBlanc K (2003). Hernias: inguinal and incisional. Lancet.

[2] Claus CMP, Rocha GM, Campos ACL (2016). Prospective, randomized and controlled study of mesh displacement after laparoscopic inguinal repair: fixation versus no fixation of mesh. Surg Endosc.

[3] HerniaSurge Group (2018). International guidelines for groin hernia management. Hernia.

[4] Saia M, Mantoan D, Buja A (2013). Increased rate of day surgery use for inguinal and femoral hernia repair in a decade of hospital admissions in the Veneto Region (north-east Italy): a record linkage study. BMC Health Serv Res.

[5] Farquharson EL (1955). Early ambulation; with special reference to herniorrhaphy as an outpatient procedure. Lancet.

[6] Mitchell JB, Harrow B (1994). Costs and outcomes of inpatient versus outpatient hernia repair. Health Policy.

[7] Deutsch N, Wu CL (2003). Patient outcomes following ambulatory anesthesia. Anesthesiol Clin North Am.

[8] Sinha S, Srinivas G, Montgomery J, DeFriend D (2007). Outcome of day-case inguinal hernia in elderly patients: how safe is it?. Hernia.

[9] Simons MP, Smietanski M, Bonjer HJ (2018). International guidelines for groin hernia management. Hernia 2018.

[10] Voorbrood CEH, Burgmans JPJ, Clevers GJ (2015). One-stop endoscopic hernia surgery: efficient and satisfactory. Hernia.

[11] Millat B, Fingerhut A, Gignoux M, Hay JM (1993). Factors associated with early discharge after inguinal hernia repair in 500 consecutive unselected patients. French associations for Surgical Research. Br J Surg.

[12] Scarfe A, Duncan J, Ma N (2018). Day case hernia repair: weak evidence or practice gap?. ANZ J Surg.

[13] Köckerling F, Lorenz R, Reinpold W (2022). What is the reality in outpatient vs inpatient groin hernia repair? An analysis from the Herniamed Registry. Hernia.

[14] Drissi F, Jurczak F, Cossa JP (2018). Outpatient groin hernia repair: assessment of 9330 patients from the French Club Hernie database. Hernia.

[15] Kark AE, Belsham PA, Kurzer MN (2005). Simultaneous repair of bilateral groin hernias using local anaesthesia: a review of 199 cases with a five-year follow-up. Hernia.

[16] Amid PK, Shulman AG, Lichtenstein IL (1996). Simultaneous repair of bilateral inguinal hernias under local anesthesia. Ann Surg.

[17] Quilici PJ, Greaney EM, Quilici J, Anderson S (2000). Laparoscopic inguinal hernia repair: optimal technical variations and results in 1700 cases. Am Surg.

[18] Ministerio de Sanidad C y BS (2021) Registro de Actividad de Atención Especializada del Conjunto Mínimo Básico de Datos (RAE-CMBD). https://www.mscbs.gob.es/estadEstudios/estadisticas/estadisticas/estMinisterio/SolicitudCMBD.htm

[19] Quan H, Sundararajan V, Halfon P (2005). Coding algorithms for defining comorbidities in ICD-9-CM and ICD-10 administrative data. Med Care.

[20] Shnaider I, Chung F (2006). Outcomes in day surgery. Curr Opin Anaesthesiol.

[21] Weyhe D, Winnemöller C, Hellwig A (2006). [(section sign) 115 b SGB V threatens outpatient treatment for inguinal hernia. Analysis of outcome and economics]. Chirurg.

[22] De Lathouwer C, Poullier (2000). How much ambulatory surgery in the World in 1996–1997 and trends?. Ambul Surg.

[23] Jarrett PE (2001). Day care surgery. Eur J Anaesthesiol Suppl.

[24] Kehlet H, Bay-Nielsen M, Danish Hernia Database Collaboration (2008). Nationwide quality improvement of groin hernia repair from the Danish hernia database of 87,840 patients from 1998 to 2005. Hernia.

[25] Guillaumes S, Hidalgo NJ, Bachero I, Juvany M (2023). Outpatient inguinal hernia repair in Spain: a population-based study of 1,163,039 patients-clinical and socioeconomic factors associated with the choice of day surgery. Updates Surg.

[26] Bringman S, Ramel S, Heikkinen T-J (2003). Tension-free inguinal hernia repair: TEP versus mesh-plug versus Lichtenstein: a prospective randomized controlled trial. Ann Surg.

[27] Metzger J, Lutz N, Laidlaw I (2001). Guidelines for inguinal hernia repair in everyday practice. Ann R Coll Surg Engl.

[28] Pallati PK, Gupta PK, Bichala S (2013). Short-term outcomes of inguinal hernia repair in octogenarians and nonagenarians. Hernia.

[29] Kurzer M, Kark A, Hussain ST (2009). Day-case inguinal hernia repair in the elderly: a surgical priority. Hernia.

[30] Hajri M, Haddad D, Zaafouri H (2022). Ambulatory hernia repair: a study of 1294 patients in a single institution. Pan Afr Med J.

[31] Palumbo P, Amatucci C, Perotti B (2014). Outpatient repair for inguinal hernia in elderly patients: still a challenge?. Int J Surg.

[32] Canet J, Raeder J, Rasmussen LS (2003). Cognitive dysfunction after minor surgery in the elderly. Acta Anaesthesiol Scand.

[33] American Society of Anesthesiologists (2021) ASA Physical Status Classification System. https://www.asahq.org/standards-and-guidelines/asa-physical-status-classification-system. Accessed 12 Mar 2022

[34] Sanjay P, Jones P, Woodward A (2006). Inguinal hernia repair: are ASA grades 3 and 4 patients suitable for day case hernia repair?. Hernia.

[35] Solodkyy A, Feretis M, Fedotovs A et al (2018) Elective true day case laparoscopic inguinal hernia repair in a District General Hospital: lessons learned from 1000 consecutive cases. 10.1155/2018/7123754. Minim Invasive Surg 2018:7123754 10.1155/2018/7123754 PMC6008672 29971162

[36] Drissi F, Gillion JF, Cossa JP (2019). Factors of selection and failure of ambulatory incisional hernia repair: a cohort study of 1429 patients. J Visc Surg.

[37] Palumbo P, Minicucci A, Nasti AG (2007). Treatment for persistent chronic neuralgia after inguinal hernioplasty. Hernia.

[38] Pavlin DJ, Chen C, Penaloza DA (2002). Pain as a factor complicating recovery and discharge after ambulatory surgery. Anesth Analg.

[39] Feliu-Palà X, Martín-Gómez M, Morales-Conde S, Fernández-Sallent E (2001). The impact of the surgeon’s experience on the results of laparoscopic hernia repair. Surg Endosc.

[40] Schmedt CG, Sauerland S, Bittner R (2005). Comparison of endoscopic procedures vs Lichtenstein and other open mesh techniques for inguinal hernia repair: a meta-analysis of randomized controlled trials. Surg Endosc.

[41] Feliu X, Clavería R, Besora P (2011). Bilateral inguinal hernia repair: laparoscopic or open approach?. Hernia.

[42] Memon MA, Cooper NJ, Memon B (2003). Meta-analysis of randomized clinical trials comparing open and laparoscopic inguinal hernia repair. Br J Surg.

[43] Rosenberg J, Friis-Andersen H, Jørgensen LN, Andresen K (2021). Variables in the Danish hernia databases: inguinal and ventral. Laparosc Surg.

[44] Madion M, Goldblatt MI, Gould JC, Higgins RM (2021). Ten-year trends in minimally invasive hernia repair: a NSQIP database review. Surg Endosc.

[45] Palser TR, Swift S, Williams RN (2019). Variation in outcomes and use of laparoscopy in elective inguinal hernia repair. BJS Open.

[46] Guillaumes S, Hoyuela C, Hidalgo NJ (2021). Inguinal hernia repair in Spain. A population-based study of 263,283 patients: factors associated with the choice of laparoscopic approach. Hernia.

[47] Andresen K, Rosenberg J (2021). Decreasing use of open procedures in elective inguinal hernia surgery. Laparosc Surg.

[48] Bittner R, Montgomery MA, Arregui E (2015). Update of guidelines on laparoscopic (TAPP) and endoscopic (TEP) treatment of inguinal hernia (International Endohernia Society). Surg Endosc.

[49] Poelman MM, van den Heuvel B, Deelder JD et al (2013) EAES Consensus Development Conference on endoscopic repair of groin hernias. Surg Endosc 27:3505–19. 10.1007/s00464-013-3001-9 10.1007/s00464-013-3001-9 23708718

[50] Simons MP, Aufenacker T, Bay-Nielsen M (2009). European Hernia Society guidelines on the treatment of inguinal hernia in adult patients. Hernia.

[51] Bittner R, Arregui ME, Bisgaard T (2011). Guidelines for laparoscopic (TAPP) and endoscopic (TEP) treatment of inguinal hernia [International Endohernia Society (IEHS)]. Surg Endosc.

[52] Hidalgo NJ, Guillaumes S, Bachero I (2023). Trends and predictors of laparoscopic bilateral inguinal hernia repair in Spain: a population-based study. Surg Endosc.

[53] Trevisonno M, Kaneva P, Watanabe Y (2015). A survey of general surgeons regarding laparoscopic inguinal hernia repair: practice patterns, barriers, and educational needs. Hernia.

[54] Whippey A, Kostandoff G, Paul J (2013). Predictors of unanticipated admission following ambulatory surgery: a retrospective case-control study. Can J Anaesth.

[55] Kingsnorth AN, Bowley DMG, Porter C (2003). A prospective study of 1000 hernias: results of the Plymouth Hernia Service. Ann R Coll Surg Engl.

[56] Van Caelenberg E, De Regge M, Eeckloo K, Coppens M (2019). Analysis of failed discharge after ambulatory surgery: unanticipated admission. Acta Chir Belg.

[57] Ngo P, Pélissier E, Levard H (2010). Ambulatory groin and ventral hernia repair. J Visc Surg.

[58] Hidalgo NJ, Bachero I, Hoyuela C (2022). The transition from open to laparoscopic surgery for bilateral inguinal hernia repair: how we did it. Langenbecks Arch Surg.

[59] Guillaumes S, Juvany M (2022). Inguinal hernia repairs performed for recurrence in Spain: population-based study of 16 years and 1,302,788 patients. Hernia.

[60] Trevisonno M, Kaneva P, Watanabe Y (2015). Current practices of laparoscopic inguinal hernia repair: a population-based analysis. Hernia.

[61] Muaddi H, Stukel TA, de Mestral C (2023). The evolving use of robotic surgery: a population-based analysis. Surg Endosc.

[62] Smith FJ, Holman CDJ, Moorin RE, Fletcher DR (2008). Incidence of bariatric surgery and postoperative outcomes: a population-based analysis in Western Australia. Med J Aust.

