# Supplementary material for: Outpatient Surgery and Unplanned Overnight Admission in Bilateral Inguinal Hernia Repair: A Population-based Study
Source: Langenbecks Arch Surg. 2024 May 27;409(1):165. doi: 10.1007/s00423-024-03358-0 (PMC11129998; doi:10.1007/s00423-024-03358-0)
Supplement: Supplementary file 1 — Supplementary Material 1 [file 423_2024_3358_MOESM1_ESM.docx]

**SUPPLEMENTARY MATERIAL**

Table S1. The ICD-10 codes used to identify specific comorbidities

|  | Charlson comorbidity index  ICD-10 codes | Elixhauser comorbidity index  ICD-10 codes |
| --- | --- | --- |
| Arterial hypertension |  | Hypertension, uncomplicated: I10.x  Hypertension, complicated: I11.x - I13.x, I15.x |
| Heart disease | Myocardial infarction: I21.x, I22.x, I25.2  Congestive heart failure: I09.9, I11.0, I13.0, I13.2, I25.5, I42.0, I42.5 - I42.9, I43.x, I50.x, P29.0 | Congestive heart failure: I09.9, I11.0, I13.0, I13.2, I25.5, I42.0, I42.5 - I42.9, I43.x, I50.x, P29.0  Cardiac arrhythmias: I44.1 - I44.3, I45.6, I45.9, I47.x - I49.x, R00.0, R00.1, R00.8, T82.1, Z45.0,  Z95.0  Valvular disease: A52.0, I05.x - I08.x, I09.1, I09.8, I34.x - I39.x, Q23.0 - Q23.3, Z95.2 - Z95.4 |
| Chronic pulmonary disease | Chronic pulmonary disease: I27.8, I27.9, J40.x - J47.x, J60.x - J67.x, J68.4, J70.1, J70.3 | Chronic pulmonary disease: I27.8, I27.9, J40.x - J47.x, J60.x - J67.x, J68.4, J70.1, J70.3 |
| Renal disease | Renal disease: I12.0, I13.1, N03.2 - N03.7, N05.2 - N05.7, N18.x, N19.x, N25.0, Z49.0 - Z49.2,  Z94.0, Z99.2 | Renal failure: I12.0, I13.1, N18.x, N19.x, N25.0, Z49.0 - Z49.2, Z94.0, Z99.2 |
| Liver disease | Mild liver disease: B18.x, K70.0 - K70.3, K70.9, K71.3 - K71.5, K71.7, K73.x, K74.x, K76.0, K76.2  - K76.4, K76.8, K76.9, Z94.4  Moderate or severe liver disease: I85.0, I85.9, I86.4, I98.2, K70.4, K71.1, K72.1, K72.9, K76.5,  K76.6, K76.7 | Liver disease: B18.x, I85.x, I86.4, I98.2, K70.x, K71.1, K71.3 - K71.5, K71.7, K72.x - K74.x,  K76.0, K76.2 - K76.9, Z94.4 |
| Diabetes mellitus | Diabetes without chronic complication: E10.0, E10.1, E10.6, E10.8, E10.9, E11.0, E11.1, E11.6,  E11.8, E11.9, E12.0, E12.1, E12.6, E12.8, E12.9, E13.0, E13.1, E13.6, E13.8, E13.9, E14.0,  E14.1, E14.6, E14.8, E14.9  Diabetes with chronic complication: E10.2 - E10.5, E10.7, E11.2 - E11.5, E11.7, E12.2 - E12.5,  E12.7, E13.2 - E13.5, E13.7, E14.2 - E14.5, E14.7 | Diabetes, uncomplicated: E10.0, E10.1, E10.9, E11.0, E11.1, E11.9, E12.0, E12.1, E12.9, E13.0,  E13.1, E13.9, E14.0, E14.1, E14.9  Diabetes, complicated: E10.2 - E10.8, E11.2 - E11.8, E12.2 - E12.8, E13.2 - E13.8, E14.2 - E14.8 |
| Obesity |  | Obesity: E66.x |
| Peripheral vascular disease | Peripheral vascular disease: I70.x, I71.x, I73.1, I73.8, I73.9, I77.1, I79.0, I79.2, K55.1, K55.8,  K55.9, Z95.8, Z95.9 | Peripheral vascular disorders: I70.x, I71.x, I73.1, I73.8, I73.9, I77.1, I79.0, I79.2, K55.1, K55.8,  K55.9, Z95.8, Z95.9 |
| Cerebrovascular disease | Cerebrovascular disease: G45.x, G46.x, H34.0, I60.x - I69.x |  |
| Rheumatic disease | Rheumatic disease: M05.x, M06.x, M31.5, M32.x - M34.x, M35.1, M35.3, M36.0 | Rheumatoid arthritis/collagen vascular diseases: L94.0, L94.1, L94.3, M05.x, M06.x, M08.x,  M12.0, M12.3, M30.x, M31.0 - M31.3, M32.x - M35.x, M45.x, M46.1, M46.8, M46.9 |
| Alcohol abuse |  | Alcohol abuse: F10, E52, G62.1, I42.6, K29.2, K70.0, K70.3, K70.9, T51.x, Z50.2, Z71.4, Z72.1 |
